# An integrative literature review on the impact of COVID-19 on maternal and child health in Africa

**DOI:** 10.1186/s12884-022-05339-x

**Published:** 2023-01-04

**Authors:** Ephraim Kumi Senkyire, Olabanji Ewetan, Dominic Azuh, Ernestina Asiedua, Rebecca White, Margaret Dunlea, Mary Barger, Magdalena Ohaja

**Affiliations:** 1 Ga West Municipal Hospital-Ghana Health Service, Amasaman, Accra, Ghana; 2 Covenant University, Ota, Ogun state Nigeria; 3 University of Ghana, Legon, Accra, Ghana; 4 Rocky Mountain University, Provo, UT USA; 5 Trinity College Dublin, Dublin, Ireland; 6 University of California, San Francisco, California USA; 7 University of Galway, Galway, Ireland

**Keywords:** Maternal and child health, COVID-19, Africa, Pandemic, Lockdowns, Health systems, Integrative review

## Abstract

Africa has the highest rates of maternal deaths globally which have been linked to poorly functioning health care systems. The pandemic revealed already known weaknesses in the health systems in Africa, such as workforce shortages, lack of equipment and resources. The aim of this paper is to review the published literature on the impact of the COVID-19 pandemic on maternal and child health in Africa. The integrative review process delineated by Whittemore and Knafl (2005) was used to meet the study aims. The literature search of Ovid Medline, CINAHL, PubMed, WHO, Google and Google scholar, Africa journals online, MIDIRS was limited to publications between March 2020 and May 2022. All the studies went through the PRISMA stages, and 179 full text papers screened for eligibility, 36 papers met inclusion criteria. Of the studies, 6 were qualitative, 25 quantitative studies, and 5 mixed methods. Thematic analysis according to the methods of Braun and Clark (2006) were used to synthesize the data. From the search the six themes that emerged include: effects of lockdown measures, COVID concerns and psychological stress*,* reduced attendance at antenatal care, childhood vaccination, reduced facility-based births, and increase maternal and child mortality. A review of the literature revealed the following policy issues: The need for government to develop robust response mechanism to public health emergencies that negatively affect maternal and child health issues and devise health policies to mitigate negative effects of lockdown. In times of pandemic there is need to maintain special access for both antenatal care and child delivery services and limit a shift to use of untrained birth attendants to reduce maternal and neonatal deaths. These could be achieved by soliciting investments from various sectors to provide high-quality care that ensures sustainability to all layers of the population.

## Key messages

Preparedness and response support to countries with high maternal and child mortality rates will be critical now than ever to reduce the negative impact of the current global pandemic.

Healthcare systems need to be strengthened to prioritize maternal and child health services during and after the COVID-19 pandemic.

This could be achieved by soliciting investments from various sectors to provide quality care that ensures sustainability to all layers of the population.

## Introduction

The specific effects of SARS-CoV-2 on maternal health include reduced accessibility to health care by women and children because of the competing needs for intensive health care services of corona virus diseases-2019 (COVID-19) patients; health care infrastructures, medical equipment, and deliverables became overstretched and inadequate to meet the needs of all patients with women and children particularly affected.

Throughout history, Africa has often faced epidemics resulting in many deaths, including Lassa fever, polio, measles, tuberculosis and human immunodeficiency virus and Ebola disease [1]. The latter was more notable in West Africa. SARS-CoV-2, commonly known as COVID-19, was first discovered in Wuhan China in December 2019 but spread globally with the first African case reported in Egypt on the 14th of February 2020. By the end of first week of March 2020, other African countries including Algeria, Cameroon, Egypt, Morocco, Nigeria, Senegal, South Africa, Togo, and Tunisia recorded their first cases with most index cases originating from Europe [2]. The World Health Organisation (WHO) declared the virus a global pandemic on the 11th of March 2020. Countries developed various related strategies including lockdowns with stringent rules.

The vulnerability of the health care systems in Africa have been exposed by past pandemics, such as Ebola, Athenian plague, Black death, the Seven-Cholera Pandemic, Justinian plague, HIV/AIDS, and Swine flu. During these pandemics there was a decline in access to healthcare during pregnancy and childbirth leading to increased risk of maternal morbidity and mortality, which further weakened the health systems [3–7]. The global measures implemented by different countries to control the spread of COVID-19 has had adverse effects on citizens. Some studies reported that in Africa the COVID-19 outbreak caused disruption and decline in maternaland child health services such as antenatal care (ANC), delivery, post-natal care (PNC), family planning and vaccinations [8–15] linked to barriers created by lack of personal protective equipment (PPE), shortage of human resources, long waiting times and others. Measures to overcome the situation caused disruptions in routine ANC as accessibility was difficult, lack of transportation, increased poverty as breadwinners were jobless, informal jobs could not thrive among other impaired circumstances. These were impediments to pregnant women trying to access to health facilities [14, 16]. Above all, health facilities were occupied with the pandemic and its related diseases. During the first wave of the pandemic, health facilities focused solely on COVID-19 to the exclusion of all other conditions hence maternal health care (MHC) services uptake fell steadily during the pandemic [13]. This decline was reported in eight sub-Saharan African countries where countries experienced MHC service disruption for at least a month with the magnitude and durations differing among countries [12].

Even though COVID-19 pandemic is not gender selective, maternal health in Africa may have been particularly affected by these measures [4, 17]. Hence this integrative review aimed to assess the impact of COVID-19 on maternal and child health service in Africa.

## Methods

### Methodology

The integrative review process delineated by Whittemore and Knafl [18] was used to meet the study aims. Integrative reviews make use of not only quantitative and qualitative studies but survey and technical reports in the grey literature that may be pertinent [18]. The method of this review followed five phases: problem identification, literature search, data evaluation, data analysis, and presentation which is a display of the results in tables and figures.

### Search strategy and selection procedure

A search of Ovid Medline, CINAHL, PubMed, WHO, Google and Google scholar, Africa journals online, MIDIRS, was performed using the following key terms: ‘COVID-19’, ‘Africa’, ‘maternal health’, ‘pandemic’, 'child health', ‘COVID-19 coronavirus’ OR ‘SARS-Cov-V-2’ AND ‘maternal health’ OR “pregnancy OR ‘perinatal’. All the studies went through the PRISMA stages, that is, identification, screening, eligibility, and inclusion [19].

### Inclusion criteria

Our search was limited to peer-reviewed research studies or grey literature which used systematic approaches to surveys or data conducted in Africa and published in English between March 2020 and May 2022. Reference lists of selected full texts were screened for additional relevant papers.

### Exclusion criteria

Conferences/updates, commentaries, or personal interviews without a clear methodology for reported data and research studies not conducted in Africa, not published in English language and not within the time frame were excluded.

### Data extraction

Two authors (E.K.S & M.O) independently reviewed all studies after duplicates were removed for inclusion and exclusion criteria. Any disagreements in assessment were resolved by discussion and mutual agreement. For each study included, we recorded the last name of author(s), year of publication, country, title, focus/aim, design/methodology, data collection method, sample size and key findings.

### Data synthesis

Thematic analysis according to the methods of Braun and Clark [20] were used to synthesize the data. The two authors independently read all included papers and coded key elements. Together they identified patterns from the codes from which key themes emerged. Literal interpretation of the data yielded the themes.

## Results

**Fig. 1:**
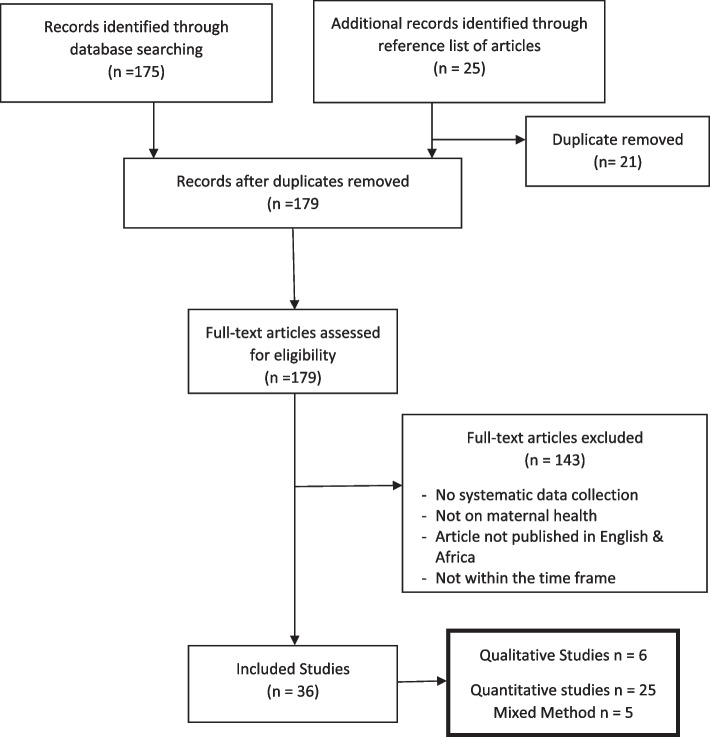
PRISMA Flow Chart

Among the 179 full text papers screened for eligibility, 36 papers met inclusion criteria (see Fig. 1). Of the studies, 6 were qualitative, 25 quantitative studies, and 5 mixed methods. (See Table 1). The studies were retrieved from 4 African regions with the greatest number from East Africa and four from several sub-Saharan African countries (see Table 2). Synthesis from the relevant studies revealed six relevant themes: effects of lockdown measures, COVID concerns and psychological stress*,* reduced attendance at antenatal care, childhood vaccination, reduced facility-based births and increase maternal and child mortality. (Table 3).

**Table 1:** Details of eligible studies

Author, year, country	Focus/Aim	Design/ Methodology	Period of data collection	Sample	Key findings
Abdisa D.K., Jaleta D.D., Feyisa J.W., et al., 2022. Ethiopia [21]	Assess the magnitude of delays in maternal health service utilization and its associated factors among pregnant women in the Ilubabor zone during the COVID-19 pandemic	Facility-based cross-sectional study	February–April 2021	402 pregnant women	Remarkable section of women experienced delays in seeking care with various factors, yet these delays were not related to pandemic fears.
Akaba G., Dirisu O., Okunade K., et al. 2022 Nigeria [22]	Explore the barriers and facilitators of access to MNCH services during the first wave of covid-19 pandemic in Nigeria	Qualitative	May–July 2020	54 study participants (service users, service providers and policymakers,)	Lack of PPE, shortage of manpower, long waiting times at the hospitals, prioritization of essential services, and lack of preparedness by health workers, were barriers to accessing MNCH services during the first wave of COVID-19. Enablers to access: COVID-19 preventive measures, community sensitization, and alternative strategies for administering immunization service at the clinics.
Asuming P.O., Gaisie D.A., Agula C., et al.,2022. Ghana [23]	Estimate the impact of Covid-19 on delivery of maternal health services using ANC attendance and delivery at health facilities as outcomes	Survey	September–November 2020	288 women (15–49 years)	Pandemic resulted in 23 and 25% reductions on facility and skilled delivery and uptake of antenatal care services respectively among women who were pregnant/ delivered before and during the pandemic period
Ateva E. 2020. Kenya [16]	Impact of COVID-19 on reproductive, maternal, and new-born health services.	Qualitative methods	April 17–May 14, 2020?	325 adolescent girls, single mothers, women, men, people living with disability, community health workers, and local administration officials.	Socioeconomic impact of the pandemic affected women/girls with reported incidences of sexual and gender-based violence, difficulties accessing health facilities and disrupted economic activities. Lack of transportation, verbal and physical harassment from law enforcing agencies when accessing health facilities.
Atim M.G., Kajogoo V.D., Amare D., et al., 2021. Uganda [24]	Identify impacts of COVID-19 on RMNCH indicators and outcomes of the HSDP in Uganda	Descriptive quantitative study	March 2020–May 2021	Government portals and review of relevant articles	3% declined in facility-based deliveries; 7.6% increase in maternal mortality. Antenatal, sexual, and reproductive health, emergency and obstetric, and postnatal care services all affected.
Balogun M., Banke-Thomas A., Sekoni A., et al., 2021. Nigeria [25]	Assess the challenges faced by women who used RMNCH services in Nigeria’s epicentre, their satisfaction with care received during the COVID-19 pandemic and the factors associated with their satisfaction.	Cross-sectional survey	September 16, 2020-October 12 2020	1241 women of reproductive age	Lockdowns and lack of transportation were barriers to accessing MHC services. Satisfaction scores for the interpersonal aspects of care were significantly lower in the PHCs and general hospitals compared to teaching hospitals.
Banke-Thomas A.,2022 Sub-Saharan Africa (Guinea, Nigeria, Tanzania, Uganda) [26]	Effect of COVID on MHC utilization in 4 sub-Saharan countries tertiary referral centres	Mixed methods	Quantitative: Mar 2019-Feb 2021. Qualitative: Semi-structured interviews July 2020-Feb 2021. Timeline data of COVID-19 epidemiology, national and hospital-level events	6 referral hospitals, 22 skill health personnel, WHO COVID-19 timeline cases	Identified 3 periods: first wave, slow wave, and second wave. MHC use lower than pre-pandemic in ¾ countries particularly 1st and 2nd wave but stable or higher than pre-pandemic during ‘slow wave’. Decreased use due to fear of infection, lack or high cost of transportation, and service closure.
Bekele C., Bekele D., Hunegnaw B.M., et al., 2022. Ethiopia [8]	Assess MNCH utilization during the first six months of the COVID-19 pandemic, as well as potential barriers and enablers of service utilization from health care providers and clients.	Mixed study design. Compared 1st 6 months pandemic to same period in prior year.	Qualitative; 2–20 November 2020. Quantitative; March 2019 to August 2020	8 health facilities (three hospitals and five health centres and interviews with 103 healthcare providers working in the MNCH units of the facilities. In addition to these, ten facility or MNCH department heads and nine women (pregnant and delivered in the time of COVID-19)	Service utilization of new family planning visits (43.2 to 28.5/month, *p* = 0.014) and sick under five child visits (225.0 to 139.8/month, *P* = 007) declined. Antenatal and postnatal care visits, facility delivery rates, and child routine immunization visits also decreased although this did not reach statistical significance. Interviews with health care providers and clients highlighted barriers to service utilization: fear of disease transmission, economic hardship, and transport service disruptions and restrictions
Bikwa Y., Murewanhema G., Kanyangarara M., et al., 2021. Zimbabwe [27]	Determine the impact of the lockdown on MCH outcomes at 2 tertiary hospitals in Harare, Zimbabwe. 2. Estimate changes in MCH interventions due to COVID on maternal, and neonatal mortality in Zimbabwe using the Lives Saved Tool (LiST)	Cross-sectional study. Retrospective study of secondary data	March–August 2020	19,835 hospital deliveries	There was diminution in the uptake of maternal health services and surged risk of dreadful maternal consequences during the lockdown
Burt J., Ouma J., Amone A., et al. 2021. Uganda [28].	Hypothesised that the early, strict lockdown which severely limited the movements of individuals in Uganda will have impacted access to services.	Observational study (quantitative)	July 2019–December 2020, July 2019–March 2020, April 2020–June 2020, July 2020–December 2020	Pregnant women	Sharp decrease in ANC attendance which remained at low level pre-covid with increased in pregnancy related problems such haemorrhage, and caesarean section
Carter ED, Zimmerman L, Qian J, et.al., 2022. Ethiopia [9]	To assess changes in RMNH intervention coverage before and during the COVID-19 pandemic using Performance Monitoring for Action Ethiopia longitudinal data	panel survey data	October and November 2019	Performance Monitoring for Action (PMA) survey	little disruption of RMNH services in Ethiopia in the initial months of the pandemic. There were no significant reductions in women seeking health services. Significant reductions in coverage of BCG vaccination observed in the COVID-19 affected cohort.
Enbiale W., Abdela S.G., Seyum M., et al., 2021 Ethiopia [29]	Assess effect of preventive COVID-19 measures on essential healthcare services in selected health facilities of Ethiopia	Comparative cross-sectional study	July 7, 2019, to July 6, 2020	Medical records of the visitors of health facilities. Data were retrieved from Health Management Information Systems	Institutional delivery, childhood immunization, antenatal care, did not vary significantly between pre-COVID-19 and during COVID-19.
Galle A., Kavira G., Semaan A., et al., 2022. DR. Congo [30].	MHC utilisation along the continuum during the COVID-19 pandemic in the Democratic Republic of the Congo, and factors associated with use of the full continuum	Cross-sectional survey	March 2020–May 2021	604 women (15–49 years) who were pregnant	Of the women who gave birth during the COVID-19 pandemic, 61% had ANC 4+ consultations, most had a skilled birth attendant (97%), and more than half (55%) had a PNC check for themselves and the newborn. One-third (36%) of women completed the continuum of maternal healthcare. Reasons for not seeking maternal care included lack of money and avoiding COVID-19 vaccination
Gebreegziabher, S.B., Marrye, S.S., Kumssa T.H., et al., 2022. Ethiopia [10]	To assess trends in selected maternal and child health services performance in the context of COVID-19 pandemic	cross-sectional data review	July 2019 to March 2021	95 government health centres, 56 public hospitals and 66 private health care facilities	Postnatal care visit, new contraceptives accepters, safe abortion care (M 87HC) and pentavalent-3 vaccination significantly decreased by 9.3%, 20.3%, 23.7 and 77.2% respectively during the first 8 months of the COVID-19 pandemic compared to the previous 8 months’ average performance.
Hailemariam S., Agegnehu W., and Derese M., 2021. Ethiopia [31]	COVID-19 related factors influencing antenatal care service uptake in rural Ethiopia	Community-based qualitative study	September 25–November 25 2020	44 pregnant women, and 9 healthcare providers	COVID-19 preventive measures, health facility related factors and individual factors were the primary cause for the decline in antenatal care service.
Kassie A., Wale A., Worke Y W., 2021. Ethiopia [11].	Evaluate indirect impact of COVID-19 on the utilization RMNCH services at government health facilities in Southwest Ethiopia, and its consequences.	comparative cross-sectional study	March–June 2019. March–June 2020	Reviewing hospital and health centres’ records	Significant reduction in mean ANC visits (943.25 vs 694.75), health facility birth (808.75 vs 619), family planning visits (4744.5 vs 3991.25), and newborn immunization (739.5 vs 528.5). In the same period rise in institutional stillbirth (14% vs 21.8%) and neonatal death (33.1% vs 46.2%).
Landrian A., Mboya J., Golub G., et al., 2022. Kenya [32].	Assess the effects of COVID-19 (ANC utilisation in Kenya and women’s reports of COVID-related barriers to ANC and correlates at the individual and household levels.	Interrupted time series design	September 2019–January 2020	1729 women, including 1189 women who delivered in healthcare facilities before the COVID-19 pandemic (from September 2019–January 2020) and 540 women who delivered during the pandemic (from July through November 2020)	Women delivering during COVID had significantly higher odds of delayed ANC initiation. Nearly half (*n* = 255/540; 47%) who delivered during COVID-19 reported that the pandemic affected their ability to access ANC
Laouan F. 2020. West Africa [33]	Effect of lockdowns on reduced income and access to basic needs esp. on women, and increased gender-based violence.	Qualitative	April 6 to 23, 2020	226 people across 12 countries	Women are struggling to access health services. Women confirm that governments and health clinics have diverted energy and attention away from Sexual Reproductive and Health and Rights (SRHR) services
Mongbo Y., Sombié I., Dao B., et al. 2021. Benin, Burkina Faso, Côte d’Ivoire, Guinea, Mali, Mauritania, Niger, Senegal, Togo [34]	Analyse the challenges and solutions for maintaining the Continuity of essential health services during the COVID-19 pandemic in Francophone West Africa.	Cross-sectional study	April 2020	18 managers of Reproductive, Maternal, Neonatal, Child and Adolescent Health (RMNCAH) and vaccination programmes	Challenges in maintaining the continuity of essential health services during the COVID were organisational, and staff related factors. Ineffective service organisation with limited equipment, and lack of standardised procedures and guidelines for diagnosing and managing coronavirus. Limited healthcare worker knowledge of COVID-19, led to postponement of vaccination programme.
Nwafor J.I., Aniukwu J.., Anozie B.O., et al., 2020. Nigeria [35]	Determine knowledge and practice of preventive measures to protect against the virus causing COVID-19 among pregnant women attending prenatal care.	Cross sectional	February–March 2020	284 Pregnant women	Although most of the study participants had adequate knowledge of the preventive measures, the level of practice of these measures remained poor.
Nwafor J.I., Okedo-Alex I.N., Ikeotuonye A.C., 2021. Nigeria [36]	Determine the prevalence and predictors of COVID-19-related depression, anxiety, and stress symptoms among pregnant women	Cross-sectional study	March 1–July 31, 2020	456 pregnant women	7.2 and 6.4% of women experienced severe and extremely severe depression respectively. 3.3 and 7.7% of participants had severe and extremely severe anxiety, respectively. Overall, 23% women had severe stress compared to 16.7% extremely severe stress.
Oladeji O., Oladeji B., Farah A.E., et al., 2020. Ethiopia [37]	Assess effect of the pandemic on the utilization of MNCH health services.	Quantitative retrospective chart review	January–March 2020, April–June 2020.	Population of 1,250,069, 127 health posts, 27 health centres and 1 regional and 1 referral hospital.	ANC visits pre-pandemic 4087.75 vs intra-pandemic 3497.75. Skilled birth attendant pre-COVID 1440.12 and intra-COVID 1131.75. Decline in both ANC and skilled birth attendant before and during the pandemic was 14.43 and 21.4% respectively.
Oluoch-Aridi J., Chelagat T., Nyikuri M.M., et al., 2020. Kenya [38]	Effects of the COVID-19 pandemic and mitigation strategies on access to health care services in informal settlements.	Qualitative methods	May–June 2020	71 women	Increase awareness of the symptoms and preventative measures for COVID-19 among women in informal settlements. Economic constraints, fear of contracting COVID among others, compounded by imposed lockdown and curfew resulted to reduced access to care. Advances in quality of care due to short-waiting times, hygiene measures, and responsive health personnel at the out-patient department were reported by most respondents.
Ombere S.O. (2021) Kenya [39]	How poor expectant mothers with low bargaining power cope during COVID-19 in Kilifi County, Kenya,	Qualitative study	June 13–July 24 2020	12 purposively selected mothers who were either expectant or had new-born babies. 5 matrons-in-charge of maternal health services and 4 traditional birth attendants.	Expectant mothers feared attending hospitals for perinatal care due to the possibility of contracting COVID-19. An increase in home deliveries with the assistance of traditional birth attendants (TBAs) who were also overwhelmed with women who sought their services.
Pires P.D., Macaringue C, Abdirazak A., et al., 2021. Mozambique [40]	Assess the impact of Covid-19 on access to maternal and children health services in Nampula and estimate Alert Community for a Prepared Hospital project sustainability	Mixed methods	March–May 2019 & 2020	Health professionals, traditional birth attendants and patients from 2 health centres and 2 hospitals	Comparing 2019 maternal health services indicators with those from 2020, the intervention group had decreases of rate of MHC and increase rate in home deliveries. The non-intervention group showed a decrease in women in first antenatal visits in first trimester. Interviews revealed that most health professionals, traditional birth attendants and patients, have adequate knowledge about Covid-19.
Semaan A., Banke-Thomas A., Amongin D., et al., 2022. Sub-Saharan Africa (Guinea, Nigeria, Tanzania, and Uganda) [41]	Assesses how maternal healthcare was provided in six referral hospitals in Guinea, Nigeria, Tanzania, and Uganda during the first year of the COVID-19 pandemic.	Mixed-methods design	qualitative data between July 2020 and February 2021 quantitative from March 2019 to February 2021 timeline data of COVID-19 epidemiology, national and hospital-level events	6 referral hospitals, 22 maternity skilled heath personnel, WHO COVID-19 timeline cases	Identified 3 periods: first wave, slow period and second wave. Skilled health personnel had challenges during the first wave due to little knowledge about COVID-19, lack of infection prevention and control training, and difficulties reaching workplace. Shortage of personal protective equipment and no rapid testing for women suspected with COVID-19 were Challenges that persisted beyond the first wave.
Shakespeare C., Dube H., Moyo S., Eet al.,2021. Zimbabwe [42]	Impact of Covid-19 and lockdown control measures on non-Covid outcomes in tertiary level maternity unit in Zimbabwe	Interrupted time series before and during COVID waves	January–March 2020, April–June 2020	All women delivered within the study period	The rate of women delivering at the hospital declined from a mean of 41.6% (SD ± 1.1) to 35.8% (SD ± 4.3) which was statistically significant, *p* = 0.03. Between January–March and April–June 2020, the mean monthly deliveries declined from 747.3 (SD ± 61.3) in the first quarter of 2020 to 681.0 (SD ± 17.6) during lockdown (*p* = 0.20). Caesarean rate declined from a mean of 29.8% (SD ± 1.7) to 28.0% (SD ± 1.7), (*p* = 0.18)
Shapira G. Ahmed T., Henriette S., et al., 2021. Sub-Saharan Africa (Cameroon, Democratic Republic of Congo, Liberia, Malawi, Mali, Nigeria, Sierra Leone and Somalia) [12]	To predict what service utilization levels would have been in March–July 2020 in the absence of the pandemic	interrupted time series design	January 2018 to February 2020	administrative systems for 63,954 facilities	All countries experienced service disruptions for at least 1 month, but the magnitude and duration of the disruptions vary. Child vaccinations were most commonly affected service and fell by the largest margin. Estimated cumulative shortfall of 328,961 third-dose pentavalent vaccinations during the 5 months in these eight countries. Decreases in MHC are less generalized, although significant declines in institutional deliveries, antenatal care and postnatal care were detected in some countries
Shikuku D., Nyaok I. K, Nyaga, L.N., et al., 2021. Kenya [43]	Determine the initial impact of COVID-19 pandemic on RMNCAH services in Kenya.	Cross – sectional design	March–June 2019 & 2020	Pregnant women, adolescent girls from 47 counties	No differences in monthly mean attendance between March–June 2019 vs 2020 for MHC services attendance pre-pandemic period and intra-pandemic period, but there were some increases in some aspect of MHC services among adolescents.
Tadesse E. 2020. Ethiopia [44]	Assess the impact of the COVID-19 on ANC utilization among pregnant women attending public facilities in Northeast Ethiopia	facility-based cross-sectional study	February 2–August 30 2020	389 pregnant women	Pandemic influenced the uptake of ANC services. Consequently, the age, residency, educational status, history of still birth, interruption, and diversion of maternity health-care service, fear of COVID-19 pandemic, and transport inaccessibility were notable factors which contributed to the low antenatal care service use by pregnant women.
Tefera B., Tariku Z., Kebede M. et al., 2022. Ethiopia [13]	Describe MNCH utilization before and during Covid-19 announcement in Ethiopia and forecast 12 months client flow, at Dire Dawa Public Health Facilities	Interrupted time series analysis	Feb 01 to March 13, 2021	Five public health centres and one public referral hospital	Total services utilization showed steady fall during the interruption point. Family planning, Institutional delivery, and child immunization sharply fell during Covid-19. PNC and child immunization dropped 30 and 16% percentage point drop respectively
Temesgen K., Wakgari N., Tefera B., et al., 2021a. Ethiopia [14]	Assess maternal health care services utilization amid the COVID-19 pandemic in West Shoa Zone, Central Ethiopia	Community-based cross-sectional study (quantitative)	July 1 – July 30 2020	844 pregnant women or those gave birth in the last 6 months before the study	Low prevalence of maternal health service utilization. Maternal educational status, distance from the health facility, monthly estimated income, fear of COVID-19 infection, permission request from husband to visit a health facility, and practicing COVID-19 prevention measures related MHC utilization.
Temesgen K., Workie A., Dilnessa T., et al., 2021b. Ethiopia [45]	Assess the impact of COVID-19 infection on RMHC services among mothers getting service in governmental health institutions of Dessie town, 2020 G.C.	Cross-sectional study design using mixed (quantitative supplemented with qualitative) method	July 1–152,020	422 women	Less decrease in ANC and delivery attendance compared to PNC attendance which decreased nearly by half. Elements that influence these services were inappropriate service delivery due to fear of health care providers, shortage of medical supplies and staff workload.
Wanyana D., Wong R.,and Hakizimana D. 2021. Rwanda [15]	Assess the change in the utilization of MNCH services during the COVID-19 outbreak.	Cross-sectional quantitative study	March–April 2019 & 2020	MCH indicators from each of the 30 districts	During the COVID-19 outbreak in Rwanda, the utilization of 15 MCH services in all four categories— ANC, deliveries, PNC, and vaccinations significantly declined.
WHO 2022. Geneva [46]	A rapid assessment of the impact of the COVID-19 pandemic on health systems and essential health services across the life course	Web-based survey	November–December 2021	233 countries	Facility-based births were reduced in 45% of the countries between November to December 2021 compared to pre-pandemic era.
Zimmerman L.A., Desta S., Karp C., et al., 2021. Ethiopia [47].	To examine the effect of COVID-19 on health facility delivery in Ethiopia.	Survey	October–November 2019, June 2020	2855 pregnant women or less than six weeks postpartum	In urban areas, a 77% reduced relative risk of delivering in a hospital during COVID. No significant differences between the pre- and COVID-19 periods within rural strata where the majority of women deliver at home (55.6%). Nationally, no change in facility births. 20.0% of urban women said COVID-19 affected where they delivered relative to 8.7% of rural women (*p*-value = 0.01).

**Abbreviations**: *ANC* Antenatal Care, *MH* maternal health care, *PNC* postnatal care, *(R) MNCH* (reproductive) maternal, newborn child health

**Table 2:** Emerging Themes

Themes	N^a^	Papers
Effects of lockdown measures.	12	Asuming et al., 2022 Ateva 2020, Atim et al., 2021, Balogun et al., 2021, Banke-Thomas 2022, Bekele et al. 2022, Galle et al., 2022, Nwafor, et al., 2020, Oluoch-Aridi et al., 2020, Shakespeare et al., 2021, Temesgen et al., 2021a, Zimmerman et al., 2021
COVID concerns and Psychological Stress	10	Temesgen et al. 2021a, Ombere 2021, Tadesse 2020, Laouan 2020, Banke-Thomas et al., 2022, Akaba et al. 2022, Abdisa et al., 2022, Mongbo et al., 2021, Semaan et al., 2022, Nwafor et al., 2021
Reduced attendance at antenatal care	13	Banke-Thomas, et al., 2022, Pires et al., 2021, Kassie et al., 2021, Atim et al., 2021, Shapira et al., 2022, Bekele et al., 2022, Oladeji et al., 2020, Burt et al., 2021, Hailemariam et al. 2021, Ombere 2021, Landrian et al., 2022, Asuming et al.,2022, Enbiale et al., 2021
Childhood vaccination	7	Cater et al., 2022, Gebreegziabher et al., 2022, Kassie et al., 2021,, Shapira et al., 2022, Tefera et al., 2022, Temesgen et al. 2021b, Wanyana et al. 2021
Reduced facility-based births	12	Ateva, 2020, WHO 2022, Shapira et al., 2022, Tefera et al., 2022, Bekele et al., 2022, Atim et al., 2021, Asuming et al., 2022, Oladeji eta al. 2020, Zimmerman et al. 2021, Kassie et al., 2021, Oluoch-Aridi et al., 2020, Enbiale et al., 2021
Increased maternal mortality.	5	Temesgen et al., 2021b, Shakespeare 2021, WHO 2022, Shikuku 2020, Bikwa et al., 2021

N^a^ = Number of Papers

**Table 3:** Country/regions of eligible studies

Country/Region	Region	No. Papers	No. Papers per Regions	
DR. Congo	Central Africa	1	Central Africa	2
Ethiopia	East Africa	13	East Africa	21
Francophone West Africa (Benin, Burkina Faso, Cote d’Ivoire, Guinea, Mali, Mauritania, Niger, Senegal Togo)	West Africa	2	Southern Africa	2
			West Africa	7
			Africa	4
Ghana	West Africa	1		
Kenya	East Africa	5		
Mozambique	East Africa	1		
Nigeria	West Africa	4		
Rwanda	Central Africa	1		
Uganda	East Africa	2		
Zimbabwe	Southern Africa	2		
Global (with focus on Africa)	Africa	4		

### Effects of lockdown measures

A primary strategy used to curtail the spread of the pandemic globally was lockdown. Although pregnant mothers were allowed to access health facilities during emergencies, they had challenges accessing the health facilities during the curfew [38]. The primary source of transportation for most women to reach birth centres is either commercial or private transport which was banned in Kenya with few public ambulances operating during curfew hours [16]. Movement restrictions and transport challenges were also identified as barriers to maternal health service uptake in Ethiopia [8, 14]. Similarly, the findings of cross-sectional survey of 1241 Nigerian women by Balogun et al., [25] and a mixed method study in Ethiopia [8] showed that lockdowns and lack of transportation were barriers to accessing MHC services.

In an effort to get citizens to comply with the lockdown rules, some African countries used the law enforcement agents who often intimidated and harassed citizens so that some were afraid of going out for what is considered essential service [48]. Among a study of four sub-Saharan countries, not only lack of transportation during epidemic peaks but the high cost of transportation limited access to healthcare [26]. According to the report of White Ribbon Alliance (WRA), at least one pregnant woman died in Uganda as a direct result of lack of transportation amid the lockdown [16]. Others observed that pregnant women could not get to the health facility due to lockdown measures and lack of transportation[21 23,24,25,26,27].

A survey conducted in Kenya revealed that women and girls reported the curtailment of economic activities affected their ability to pay for services and therefore limited healthcare access [16]. This was similar in Ethiopia where economic suffering prevented women from being able to pay for transportation [8]. A cross-sectional study of 1600 pregnant women in Democratic republic of Congo between March 2020–May 2021 identified similar issues [30]. This study showed that lack of money along with vaccine hesitancy were some of the reason’s women did not access MHC services [30].

The lack of access to health services due to lockdown measures compelled pregnant women to acquire more knowledge of the pandemic and preventive measures. The increased level of knowledge was attributed to media campaign, which aimed to educate citizens on preventive measure to reduce transmission of the virus [35]. Pregnant women’s knowledge and practice of preventive measures against COVID-19 in a low-resource African setting deduced that whilst awareness level of most respondents concerning preventive measures was sufficient, it was observed that the level of practice of these preventive measures was inadequate [35]. On the other hand, a study in Ethiopia found women who practiced infection preventive measures and wore face masks were 2–5 times more likely to access MHC than those who did not [14].

### COVID concerns and psychological stress

Several studies documented women’s concerns to access MCH services for themselves and their children due to concerns of acquiring SARS-CoV-2 [14, 33, 39, 44]. A rapid gender analysis in West Africa found a reduction in access to maternal health services due to doubt about health of healthcare workers and the fear of succumbing to COVID-19 [33]. Temesgen and colleagues [14] identified fear of contacting SAR-CoV-2 as a significant barrier in maternal health service uptake in Ethiopia. This was echoed by another four-country study by Banke-Thomas et al., [26]. Several studies specifically cited women’s concerns about the lack of PPE for both patients and staff [14, 31]. A Nigerian qualitative study identified barriers to accessing MHC included both women’s concerns about lack of PPE and shortage of manpower [22]. Interestingly, a study from Southwest Ethiopia among 402 pregnant women identified important delays in seeking care but fear of contagion or other aspects of the pandemic were not among them [21].

Lack of adequate staffing was due to a combination of lack of personnel out sick due to COVID or concerns about contacting the disease [22, 34] and lockdown effects of limiting transportation and economic activity. This resulted in lack of preparedness by health workers, prioritization of essential services, and long wait times at the hospitals all of which were identified as barriers to accessing maternal, newborn and child health services during the first wave of COVID-19 [22]. Similarly mixed method study among skilled healthcare providers in six referral hospitals in Guinea, Nigeria, Tanzania, and Uganda to assesses how maternal healthcare was rendered identified inadequate knowledge about COVID-19, lack of infection prevention and control training, and difficulties reaching workplace as barriers in providing MHC services [41]. However, shortage of PPE and lack rapid testing for women suspected with COVID-19 were challenges that continued past the first wave [41].

Effects of the pandemic on psychological health have been well documented worldwide. Nwafor and colleagues [36] measured depression and anxiety in 456 pregnant women attending antenatal care in Nigeria during the ongoing pandemic. The rate of moderate or more severe depression was 27 and 22% rated as having moderate to severe stress.

### Reduced attendance at antenatal care

Access to high quality ANC potentially jeopardized the safety of parturient women during pregnancy during episodes of high prevalence of COVID 19 cases. COVID-19 caused distortion in health care services leading to non-regular attendance during ANC in rural and urban areas [26]. A mixed method study in eight health facilities in Ethiopia comparing the service utilisation trends in the first 6 months of COVID-19 with the corresponding time and data points of the preceding year showed reduced ANC visits (208.9 to 181.7/month, *p* = 0.433) and under 5 visits (225.0 to 139.8/month, *p* = 0.007) [8]. An assessment of effect of the pandemic on the uptake of MHC in Somali region of Ethiopia, through a retrospective chart review revealed that the mean number of ANC pre-pandemic was 4088 and intra-pandemic was 3498., reflecting a 14% decline in ANC attendance [37] . This decline in ANC visits was corroborated by a national study using reproductive, maternal, and newborn health services data from governmental health facilities in Ethiopia during a similar time period [11]. Similarly, there was sharp decreased in antenatal clinic attendance in Uganda which was already low pre-covid with an increase in pregnancy related problems such haemorrhage, and caesarean section [28]. This was particularly true during the first phase between March and June not only in Uganda but also Nigeria [26]. Similar sharp decreases in ANC visits were observed in eight sub-Saharan countries [12].

Reasons for the low ANC attendance recorded during the COVID-19 period included inappropriate service delivery, pandemic preventive measures, shortage of medical supplies, and staff workload [31, 45]. As documented by previous themes, pregnant mothers feared attending ANC would increase their probability of contracting COVID-19 [39]. Similarly, a facility-based cross-sectional study conducted between February and August 2020, among 389 pregnant women found that diversion of maternity health-care service to COVID-19 related services, fear of COVID-19 infection, and transport inaccessibility were notable factors which contributed to the low antenatal care service use by pregnant women in Ethiopia [44].

An interrupted-times series analysis compared 1189 women who delivering in healthcare facilities before the COVID-19 pandemic (September 2019–January 2020) and 540 women who delivered during the pandemic (July through November 2020) [32]. The analysis found that women who delivered during COVID-19 had a 72% higher odds of delayed ANC commencement compared to pre-pandemic data (OR 1.72, 95% CI 1.24 to 2.37). Moreover, 47% of women who delivered during COVID-19 stated that the pandemic affected their capability to access ANC [32]. A study using a similar design in Ghana, documented a 25% reduction in women attending at least 4 ANC visits during the pandemic compared to pre-pandemic [23]. A similar pattern was identified by Banke-Thomas and colleagues [26] among four sub-Saharan countries but mostly during the first wave (March to June 2020) and the second wave (from last quarter of 2020 through Feb 2021). Conversely, in a comparative study in Ethiopia to compare effect of the COVID-19 pandemic preparation and response on essential health services in primary and tertiary healthcare facilities found no significant variation in ANC visits. The study reported mean ANC visits of 910 pre-COVID and 941 during the pandemic period. However, there was approximately 38% decline of the annual mean visits in April (572) with an increase in visits between May and June by 128% (114) [29] indicating delayed care.

### Childhood vaccination

The COVID-19 pandemic has had an impact on childhood vaccination. There was significant decline in Bacillus Calmette-Guerin (BCG) and pentavalent-3 vaccinations during the early period of the pandemic in Ethiopia [9, 10]. Pires et al., [40] investigated whether increased community awareness of the prepared state of a general hospital and health centre for COVID-19 in Mozambique would have a differential effect on use of maternal and child health services compared to 2019 and a comparable hospital and health centre outside the intervention campaign area. There was no effect from the community awareness program and both intervention and control settings experienced as decrease in first antenatal visits in the first trimester of 26 and 12%, respectively. There was also a decline on the percentage of children completing their vaccinations of 16–18% [40]. Similarly, there was sharp 16% decrease in total newborn vaccinations in an interrupted time series study in Ethiopia comparing pre-pandemic and pandemic time periods (739.5 compared to 528.5) [11, 13]. Notably, the utilization of MHC services and uptake of vaccinations declined during COVID-19 outbreak in Rwanda [15] and Uganda [24]. A similar interrupted time series study in eight sub-Saharan countries found child vaccinations were the most affected health service [12]. An estimated cumulative deficit of 328,961 third-dose pentavalent vaccinations during the 5 months in these countries was recorded [12].

### Reduced facility-based births

The easiest metrics to assess the COVID pandemic’s effect on access to MCH services is the number of births in facilities with skilled birth attendants. Most practically, the government issued curfews may have limited pregnant women’s power to travel and access health services during labour. Similarly, fear of being harassed by security agents reduced health facility births [16]. A WHO survey of 11 African countries showed facility-based births were reduced in 45% of the countries between November to December 2021 compared to a comparable time period pre-pandemic [46]. Additionally, two interrupted time series studies in Ethiopia and among eight other sub-Saharan Africa countries found significant decline in facility delivery births [12, 13].

However, facility birth decline was not universal. Bekele and colleagues reported reduced facility delivery rates in Ethiopia which was not statistically significant (90.7 to 84.2/month, *p* = 0.776) [8] and Enbiale and colleagues [29] also in Ethiopia did not find a significant decline. A cross-sectional study at Mpilo - Zimbabwe Central Hospital compared routine monthly maternal and perinatal statistics three months before and after COVID-19 emergency measures were implemented. The study did not find a significant decrease in births but there was a statistically significant decline in the proportion of births among women booked to deliver at the hospital from a mean of 41.6% (SD ± 1.1) to 35.8% (SD ± 4.3) (*p* = 0.03) [42]. The magnitude of decline in facility births ranged from a low of 3% in Uganda [24] to 15%-point decrease in 5 regions of Ghana [23]. Studies in both Somalia and Ethiopia recorded over a 20% decrease in skilled birth attendant births [11, 37]. A study in six regions of Ethiopia covering 91% of the population, found 77% reduced risk of facility-based births during the pandemic compared to pre-pandemic among urban women but no difference in facility-based births among rural women [47]. This resulted to a rise in facility stillbirth (14% vs 21.8%) and neonatal death (33.1% vs 46.2%) [11]. The facility delivery disparity rate seen here from the narrative of Ethiopia could be attributed to cultural practices such encouraging home delivery, fear and anxiety associated with the pandemic, misinformation by media, lockdowns, health system resilience, and no support from healthcare workers [8, 13, 29].

### Increased maternal and child mortality

There is some evidence that delays in care due to numerous issues related from COVID-19 increased maternal mortality. A WHO survey among 11 African countries identified an average 16% increase in health facility maternal deaths February to May 2020 which decreased to an 11% increase in 2021 [46]. Correspondingly, secondary data analysis from government portals in Uganda from 2019/20 financial year supplemented by analysis of relevant article published up May 2021 found maternal mortality increased by 7.6% during COVID-19 period [24]. On the other hand, a study using the Kenyan Health Information System comparing 2019 data to March to June 2020 data did not show any differences in the maternal mortality ratio except for noting a trend in an increase of adolescent maternal deaths from 6.2 to 10.9% [43].

There is some evidence of increased maternal morbidity during COVID restrictions. Bikwa et al. [27] carried out a retrospective review of maternal audit to determine the impact of COVID-19 on maternal and perinatal outcomes in Harare, Zimbabwe. They compared data from March–August 2020 with data from March–August 2019 which showed a decreased uptake of maternal health services in 2020 and increased maternal morbidities of uterine rupture possibly due to 30% decreased odds of caesareans.

Perinatal and neonatal outcomes also may have worsened. Bikwa et al.’s [27] study in Harrare also found increased odds of both stillbirths and neonatal deaths. Kassie and colleagues [11] identified a sharp rise in facility stillbirth (14% vs 21.8%) and neonatal deaths (33.1% vs 46.2%) in their study in Ethiopia. The study done in Mpilo-Zimbabwe Central Hospital did not find an increase in early neonatal deaths [42].

## Discussion

Our integrative review identified significant effects of the pandemic on access to and the quality of care available to pregnant women in all major regions of Africa as well as delayed vaccinations among children. Pregnant women not only had trouble accessing services because of transportation restriction and high cost of transport but also because maternal health services were curtailed due to lack of healthcare workers, either due to illness, lack of PPE, or shifting personnel to care for COVID-infected patients [49]. The lack of appropriate healthcare due to the pandemic is even more important given the low rates of vaccination in Africa and the higher rates of adverse maternal outcome due to preeclampsia [50], gestational diabetes [51] and possibly thrombotic events as a consequence of additional risk factors including pregnancy itself and surgical procedures performed during labour or after delivery [52].

There is evidence that delayed or curtailed care resulted in increased maternal morbidity and mortality as well as increased neonatal morality [11, 16, 24, 27, 43, 46]. The full effect of maternal lives lost from the shift from facility to home births may not yet be appreciated due to the slow reporting for maternal deaths at home [46].

Our results on the effects of COVID on worsening maternal health echo the findings of a larger global systematic review of both low- and high-income countries with the exception of preterm births which only increased for high income countries [53]. However, unlike that review of mixed income countries which did not find significant differences in maternal morbidities or stillbirths, our review found evidence that weakened health facilities and changes to usual care from lockdown measures had an adverse effect on maternal and neonatal outcomes among some African countries. A review by Kotlar et al., [54], conclude that on one hand, when compared with non-pregnant women, risk of COVID-19 infection was not higher among pregnant women. On the other hand, pregnant women with COVID-19 symptoms are more likely to experience adverse outcomes than their non-pregnant counterparts [54].

Both high- and low-income countries have identified worsening of people’s mental health during the pandemic [55]. Several studies have documented the pandemic may have had a differential impact on women due to not only loss of employment but the burden of other responsibilities, such as running the household, general caring for children with the added role of taking on educational tasks [55]. A systematic review related to mental health effects of COVID on pregnant and lactating women found from studies in middle- and high-income countries, high rates of anxiety, depression, and social dysfunction [54, 56]. This echoes the findings in the single study in our integrative review that addressed the mental health effects among pregnant and postpartum women in Nigeria which documented a rate of severe depression of 22% and moderate to severe anxiety of 27% [36]. Although these authors did not report a comparable baseline rate of depression among Nigerian pregnant women, a global systematic review reported a baseline rate of 15% indicating a potentially significant effect of the COVID-19 epidemic on pregnant women’s mental health [57]. Socially, stigmatisation is a vital facet of infectious diseases such as leprosy, severe acute respiratory syndrome and AIDS [58]. In a study by Strong and Schwartz [59], expectant women were stigmatised by denying them access to services during Ebola epidemic by healthcare workers for the fear of being infected with the disease.

An important finding from Almeida et al., [55] on the differential effect of COVID on women was the importance of social support as a mediator in dampening the effects of the pandemic on women’s mental health. Some governments in African countries failed to put in place adequate measures to bolster social support to mitigate the negative fallout of the lockdown measures, such as loss of daily income by low-income families in the informal sector, psychological and social effects on women and children, security threats to lives and properties, and other criminal activities [16, 35, 38]. An important lesson for the future is that governments need to better analyse the costs and benefits of different actions in preparing for the next epidemic. Also, better methods of clear and transparent messaging are essential so that people do not lose trust in what they are hearing from government messaging.

The COVID-19 lockdown showed that the measures put in place was not fool proof as other ingredients should have been integrated into the arrangements. Most of the studies reported that measures to prevent and/or decrease the spread of SARS-CoV-2 infection such as curfews and inflexible law enforcement agents, limited access to needed medical care, transport curtailment, total closure of government offices and private businesses, closure of airports were not welfare considered and people centred in implementation [14, 16, 38]. Additionally, the pandemic revealed already known weaknesses in the health systems in Africa, such as workforce shortages, lack of equipment and resources – particularly PPE, and lack of suitable training of personnel. The needed shift of resources to caring for very ill COVID patients meant fewer resources for pregnant and labouring women [22, 49]. Recommendations to deal with epidemic related issues in the future include a need to develop plans ahead of time, methods to limit exposure of health personnel through adequate training in the use of PPE and adequate availability of PPE but also conserving PPE to be used only when needed [49]. Additionally, there needs to be a focus on maintaining health personnel’s well-being through the provision of resources, such as childcare and meal preparation, as well as psychological support to manage the stress of working during such as crisis [49]. Also, governments should create a buffer fund with legislative support to enable adequate and timely provision of financial and social and health support services to vulnerable groups and low-income families that depend on daily income [60, 61]. Governments need to improve funding of primary health care and introduce robust mechanisms to respond to health emergencies and crises [62, 63]. Funding and vaccine policies are needed for pregnant women in Africa because there is evidenced showing post-infection maternal SARS-CoV-2 humoral immunity decreases quickly during pregnancy, resulting in small or vague protective antibody for a substantial proportion of pregnant women. Moreover, a single boosting dose vaccine induced a strong rise in protective antibody for mother and newborn [64].

One of the most difficult issues is how to recruit and train needed personnel during an epidemic since unlike high resourced countries with many more health providers per population, in general African countries are already understaffed. A fuller examination of the efficiency and cost of the approach adopted by some countries of recruiting and training unemployed health workers and incentivizing public health workers is needed [65].

Several studies confirmed a decrease in ANC and skilled birth attendants leading to an increase in pregnancy-related problems and decrease in immunizations [23, 26, 28, 32, 38, 44]. Reduced attendance at ANC was a reality due to the public health concerns of the disease being spread among groups of people. However, ANC plays a crucial role in maintaining the health of pregnant women and their foetuses. One option might be establishing “mid-point clinics” to ensure proximity to health facilities but overcomes the challenges of inadequate space at health facilities, and reduces the burden of transportation and other lockdown measures that served as hindrances to accessing health facilities during an emergency.

### Limitations

This focussed mainly on papers published in the English language. Some papers from Francophone and Lusophone African not publish in English with no available English translation were automatically excluded. As already noted, the review included papers published between March 2020 and May 2022 because the first recorded COVID case in Africa was on the 14th of February 2020. We acknowledge that only paper [36] made reference to mental health in this review. However, it is important to note this was an unintentional discovery. Maternal mental health was not included as a search term for this review.

## Conclusion

The already existing limited access to quality maternal healthcare was exacerbated by the pandemic. While it is important to understand the extent to which women and their infants are susceptible to COVID-19, it is also crucial to comprehensively understand how the pandemic influenced other factors which affected access to quality and safe care, either directly or indirectly. Efforts need to be made to ensure that basic maternal health needs of women such as access to up-to-date information, quality care, and availability of transportation among others are not ignored any at time. Healthcare systems need to be strengthened to prioritize maternal and child health services during and after the COVID-19 pandemic. This could be achieved by soliciting investments from various sectors to provide high-quality care that ensures sustainability to all layers of the population. As noted by Ameyaw et al., [66], training and motivating healthcare providers in the use of remote approaches such as telemedicine and phone-based referral network could go a long way in securing women’s confidence during a pandemic period.

## Abbreviations

WHO: World Health Organisation
AIDS: Acquired Immunodeficiency Syndromes
ANC: Ante-Natal Care
BCG: Bacillus Calmette-Guerin
COVID-19: Corona Virus Diseases-2019
HIV: Human Immunodeficiency Virus
MHC: Maternal Health Care
OECD: Organisation for Economic Co-operation Development
PNC: Post-Natal Care
PPE: Personal Protect
ive: Equipment
WHO: World Health Organisation
WRA: White Ribbon Alliance
